# Prevalence and Factors Associated With Obesity Among School-Going Adolescents of Guwahati City

**DOI:** 10.7759/cureus.114041

**Published:** 2026-08-06

**Authors:** Arunima Sarmah, Achyut Chandra Baishya, Mehzabin Haider Hazarika, Jutika Ojah, Manas Kalita

**Affiliations:** 1 Community Medicine, Gauhati Medical College and Hospital, Guwahati, IND; 2 Community Medicine, Nalbari Medical College and Hospital, Nalbari, IND; 3 Community Medicine, Pragjyotishpur Medical College and Hospital, Guwahati, IND

**Keywords:** body mass index, contributing factors, overweight, prevalence, trans fats

## Abstract

Background

Adolescent obesity is a growing public health concern in urban India, but region-specific data from Guwahati are limited. This study estimated the prevalence of obesity and the factors associated with it among school-going adolescents in Guwahati.

Methods

A community-based cross-sectional study was conducted among 705 school-going adolescents, including 368 (52.2%) boys and 337 (47.8%) girls, aged 10-19 years, selected from government and private schools of Guwahati through stratified random sampling over one year. Anthropometric measurements were taken, and a predesigned and pretested semi-structured schedule was used to collect information on socio-demographic characteristics, dietary habits, physical activity, screen time and substance use. BMI was classified using standard BMI criteria, with obesity defined as BMI ≥25.0 kg/m². Obesity versus non-obese status was the primary outcome. Its association with categorical variables was assessed using the Chi-square test. The independent t-test was used to compare mean values of continuous anthropometric variables (height, weight, body mass index, waist circumference, hip circumference, and waist-to-hip ratio) between male and female adolescents. Because no adolescent in the lowest screen-time category was obese, a dose-response trend test (a trend odds ratio from grouped logistic regression, checking whether obesity risk rose steadily with increasing screen time) was used in place of a category-specific odds ratio. A p-value<0.05 was considered statistically significant.

Results

Among the 705 adolescents, the prevalence of obesity was 8.1% (57/705); for descriptive context, 112 (15.9%) were overweight, and 130 (18.4%) were underweight. Obesity (versus all non-obese adolescents, i.e. underweight, normal weight, and overweight combined) was significantly associated with regular physical activity (p<0.001; OR 5.14, 95% CI 2.94-8.97), screen time (dose-response trend OR 1.87 per one-category increase, 95% CI 1.43-2.43, p<0.001), fruit consumption (p = 0.032; OR 1.81, 95% CI 1.05-3.14), vegetable consumption (p = 0.025; OR 1.89, 95% CI 1.07-3.31), and trans-fat consumption (p = 0.021; OR 1.98, 95% CI 1.10-3.56). Adolescents with lower levels of physical activity, longer daily screen time, infrequent fruit and vegetable consumption, and frequent trans-fat consumption had a higher prevalence of obesity. No statistically significant association was observed between obesity and age, gender, or socioeconomic status (p>0.05).

Conclusion

The substantial burden of obesity among urban adolescents in Guwahati reflects India's ongoing nutrition transition. Interventions targeting screen time reduction, increased physical activity, and healthier dietary habits may help address this emerging epidemic.

## Introduction

Adolescence, typically spanning ages 10 to 19, marks the transitional stage between childhood and adulthood. It is characterized by significant physical, emotional, and behavioural changes. Challenges faced during this critical developmental period can lead to long-term health issues, including increased risks of chronic illnesses and mortality [1]

There are approximately 1.3 billion adolescents globally, making up one-sixth of the world's population, and early 90% live in low- and middle-income countries [2]. India has the largest adolescent population in the world, with 253 million, and every fifth person is between 10 and 19 years old [3]. Adolescence is characterized by a distinct set of health challenges, encompassing undernutrition and obesity, mental disorders, early and unintended pregnancy, HIV and other sexually transmitted infections, violence, road traffic injuries, and substance use [1].

Malnutrition in childhood - spanning undernutrition, micronutrient deficiencies, and overweight/obesity - represents a triple burden that is especially pronounced in low- and middle-income countries, undermining health and limiting children's long-term developmental potential [4]

India is witnessing rapid economic development and nutritional transition, which is linked with a change in the eating habits and physical activity of people, especially among children [5]. Rapid urbanization and changing lifestyle patterns, characterized by increased consumption of energy-dense foods, sedentary behaviour, prolonged screen time, reduced physical activity, and changing family environments, have substantially contributed to the growing burden of overweight and obesity among children and adolescents [6]

According to WHO 2025 statistics, worldwide adult obesity has more than doubled since 1990, and adolescent obesity has quadrupled. Over 390 million children and adolescents aged 5-19 years were overweight in 2022, including 160 million who were living with obesity [7]. According to the World Obesity Atlas 2026, the prevalence of overweight and obesity (high BMI) among children and adolescents (5-19 years) increased from 14.6% in 2010 to 20.7% in 2025. India ranks second globally for children living with overweight and obesity, with approximately 41 million children and adolescents (5-19 years) with high BMI and 14 million with obesity in 2025 [8]

Regional data from Assam reveal considerable variation in the reported burden of adolescent obesity. A school-based study among 465 students of classes 8 to 10 in Guwahati city reported a comparatively low prevalence of overweight and obesity (5.4% and 2.8%, respectively), significantly higher among non-vegetarians and those with irregular breakfast habits, infrequent fruit consumption, frequent fast-food, junk-food, and carbonated-drink consumption, limited outdoor play, and vehicular commuting to school [9]. By contrast, a study among 1096 early adolescents aged 10-14 years in Dibrugarh, Assam, using both body mass index and body fat percentage criteria, reported a considerably higher prevalence of overweight and obesity by BMI of 20.9% and 10.2%, respectively [10]. Alongside this emerging burden of excess weight, undernutrition remains prevalent among adolescents in the region: a study of 400 adolescent girls in the urban slums of Guwahati found that one in five had a BMI below the 5th percentile, illustrating the double burden of malnutrition that characterizes many transitioning urban Indian populations [11].

These regional studies, however, are largely restricted to specific age bands, a single gender, or peri-urban and slum populations, and none provide contemporary, city-wide prevalence estimates spanning the full 10-19 year adolescent age range across both government and private schools of Guwahati. Given this gap and the documented urban predisposition to factors associated with obesity, this study was undertaken to estimate the prevalence of obesity and to determine variables associated with obesity among school-going adolescents living in Guwahati city.

This article was previously presented as a conference abstract at the 51st Annual National Conference of the Indian Association of Preventive and Social Medicine (IAPSMCON 2024), hosted by the Department of Community Medicine, Kasturba Medical College, Mangalore, India, from February 8-10, 2024.

## Materials and methods

A community-based cross-sectional study was conducted among adolescents studying in high schools and higher secondary schools of Guwahati city, Assam, over a one-year period from November 2023 to October 2024. The study included adolescents aged 10-19 years studying in classes IV to XII. Adolescents aged 10-17 years who provided written informed assent along with written informed consent from their parents or legally authorized representatives (LARs), and adolescents aged 18-19 years who provided written informed consent, were included in the study. Students who declined to participate or were absent on the day of data collection were excluded.

The sample size was calculated using the formula $n = \frac{4pq}{L^2}$, considering the prevalence (*p*) of obesity among adolescents to be 14% [12], where *q = 1 - p = 0.86*. With an allowable error (*L*) set at 20% of the anticipated prevalence (*L = 0.20 × 0.14 = 0.028*) and a 95% confidence level, the calculated minimum sample size was 614. After adding a 10% non-response rate, the final sample size was estimated to be 675 participants

The sampling frame comprised all eligible schools in Guwahati city. A comprehensive list of schools was obtained from the Inspector of Schools, Kamrup (Metropolitan) District and included all government and private schools affiliated with both the state and national education boards. Overall, there were 47 government high schools, 24 government higher secondary schools, and 256 private higher secondary schools. This complete list of schools served as the sampling frame for the study.

A multistage stratified random sampling technique was employed. In the first stage, all schools were stratified into government and private schools. Government schools were further stratified into high schools and higher secondary schools. From these strata, two government high schools, one government higher secondary school, and four private higher secondary schools were selected using simple random sampling by the lottery method.

Following school selection, students were selected by simple random sampling using the lottery method from the class registers. A total of 705 students were included in the study, of whom 345 were selected from government schools and 360 from private schools. Students were selected proportionately from each eligible class to ensure adequate representation across all grades.

Among the government schools, 345 students were selected from two government high schools and one government higher secondary school. Each government high school contributed 105 students. As these schools had seven eligible classes (Classes IV to X), 15 students were selected randomly from each class using the class register (15 × 7 = 105). The government higher secondary school contributed 135 students. Since it included nine eligible classes (Classes IV to XII), 15 students were selected randomly from each class (15 × 9 = 135).

Among the private schools, 360 students were selected from four private higher secondary schools, with 90 students recruited from each school. Each private higher secondary school had nine eligible classes (Classes IV to XII), and 10 students were selected randomly from each class using the class register (10 × 9 = 90). Thus, the final study sample comprised 705 students selected from two government high schools, one government higher secondary school, and four private higher secondary schools.

Prior to data collection, ethical approval was obtained from the Institutional Ethics Committee of Gauhati Medical College and Hospital, Guwahati (Approval No. 190/2007/Pt-II/Oct/2023/3; Date of Approval: 13 October 2023). Permission to conduct the study was obtained from the principals of the selected schools after explaining the objectives of the study. Written informed consent was obtained from parents or guardians for participants aged 10-17 years, along with written assent from the adolescents. Participants aged 18-19 years provided written informed consent before enrolment. Confidentiality and anonymity of the participants were maintained throughout the study.

Data were collected using a predesigned and pretested semi-structured schedule. Sociodemographic information for participants aged 10-17 years was obtained through questionnaires completed by parents or guardians, and incomplete responses were clarified through follow-up telephone calls. Information from participants aged 18-19 years was collected directly through face-to-face interviews. 

All questionnaires and schedules were reviewed for completeness at the time of data collection. Incomplete responses were clarified immediately with the participants or their parents/guardians wherever feasible. Consequently, there were no missing data for the variables included in the final analysis. If a selected student was absent on the day of data collection, the next eligible student from the same class attendance register was enrolled to maintain the required sample size. This replacement was restricted to students from the same sampling stratum (school and class), thereby preserving the sampling framework and minimizing disruption to the sampling process.

Anthropometric measurements were obtained using standard procedures. Height was measured to the nearest 0.1 cm using a portable stadiometer with participants standing barefoot in the Frankfurt plane, while weight was measured to the nearest 0.1 kg using a calibrated digital weighing scale with participants wearing light clothing and no footwear. Body mass index (BMI) was calculated as weight in kilograms divided by the square of height in metres (kg/m²).

Operational definitions for physical activity, screen time, dietary variables, and substance use are provided as a supplementary table in the Appendices. Physical activity, fruit/vegetable/trans-fat consumption, and substance use were each dichotomized as “Yes”/“No” based on established or study-defined thresholds; screen time was captured as a five-level ordinal variable. The schedule was pretested among 70 students in one non-study school for clarity and comprehensibility; no items were revised. Formal validity or reliability testing (e.g., content validity review, Cronbach’s alpha) was not undertaken.

Nutritional status was classified according to the Asia-Pacific BMI classification as underweight (<18.5 kg/m²), normal weight (18.5-22.9 kg/m²), overweight (23.0-24.9 kg/m²), obese class I (25.0-29.9 kg/m²), and obese class II (≥30.0 kg/m²) [13]. The Asia-Pacific BMI classification was used because it is more suitable for Asian populations, who tend to have a higher body fat percentage and greater risk of obesity-related health problems at lower BMI values than Western populations. The primary outcome for this study was obesity, defined as a BMI ≥25.0 kg/m² (combining Asia-Pacific obese class I and class II). For all bivariate analyses of factors associated with obesity, participants were grouped as obese (≥25.0 kg/m²) versus non-obese, with the non-obese group comprising underweight, normal weight, and overweight participants combined.

Data were entered into Microsoft Excel (Microsoft Corporation, Redmond, USA) and analysed using IBM SPSS Statistics version 25.0 (IBM Corp., Armonk, USA). Descriptive statistics were used to summarize participant characteristics and are presented as frequencies and percentages. Associations between obesity status (obese versus non-obese) and independent variables were assessed using the Chi-square test, while the independent samples t-test was used to compare the mean values of continuous anthropometric variables (height, weight, body mass index, waist circumference, hip circumference, and waist-to-hip ratio) between male and female adolescents. For screen time, no adolescent in the lowest exposure category was obese, so a category-specific odds ratio could not be calculated for that group; instead, a dose-response trend test was applied, using a trend odds ratio derived from grouped logistic regression across the ordered screen-time categories to check whether the odds of obesity rose steadily as daily screen time increased. A p-value of <0.05 was considered statistically significant.

## Results

A total of 705 school-going adolescents (368 boys and 337 girls) aged 10-19 years were included in the study, with participants distributed across the three predefined age groups. Most adolescents belonged to nuclear families and were from middle- or upper-middle socioeconomic classes. The majority resided with their families rather than in hostel accommodation (Table 1).

**Table 1:** Distribution of adolescents according to their sociodemographic and family characteristics (N=705) ^*^Socioeconomic class assigned using the Modified B.G. Prasad classification (2024 revision) [14], which grades households into five classes (I-V) based on per-capita monthly family income adjusted to the current Consumer Price Index.

Characteristic	n	%
Age group		
10-12 years	176	25.0
13-15 years	219	31.0
16 years and above	310	44.0
Gender		
Male	368	52.2
Female	337	47.8
Type of family		
Nuclear	520	73.8
Joint	185	26.2
Living status		
With family	607	86.1
Hostel	98	13.9
Socioeconomic class*		
Class I (Upper)	140	19.9
Class II (Upper middle)	187	26.5
Class III (Middle)	221	31.3
Class IV (Lower middle)	106	15.0
Class V (Lower)	51	7.3
Total	705	100.0

The anthropometric characteristics of the participants according to gender are presented in Table 2. Mean values of height, weight, BMI, waist circumference, hip circumference, and waist-to-hip ratio did not differ significantly between male and female adolescents (all p>0.05). 

**Table 2:** Distribution of adolescents according to their anthropometric measurements (N=705) ^*^SD= Standard Deviation. ^†^BMI= Body Mass Index. ^‡^Waist–hip ratio = waist circumference (cm) / hip circumference (cm). ^§^Independent samples t-test used for all comparisons.

Characteristic	Male (Mean ± SD^*^) (n=368)	Female (Mean ± SD^*^) (n=337)	t-test statistics^§^	p-value
Height (cm)	150.5 ± 10.7	151.1 ± 10.4	-0.755	0.451
Weight (kg)	50.3 ± 11.3	50.1 ± 11.2	0.236	0.814
BMI^†^ (kg/m²)	22.0 ± 4.1	21.8 ± 4.4	0.623	0.534
Waist circumference (cm)	80.1 ± 11.4	80.6 ± 11.7	-0.574	0.566
Hip circumference (cm)	89.6 ± 11.1	90.0 ± 10.8	-0.485	0.628
Waist-to-hip ratio^‡^	0.90 ± 0.12	0.90 ± 0.11	0.000	1.000

As shown in Table 3, the prevalence of obesity was 8.1% (57/705). For descriptive context, 406 (57.6%) participants had a normal BMI, 130 (18.4%) were underweight, and 112 (15.9%) were overweight; these overweight and underweight adolescents were classified as non-obese for all subsequent bivariate analyses of factors associated with obesity, indicating a substantial burden of obesity in the study population.

**Table 3:** Distribution of adolescents by reclassified BMI status (N = 705) *Non-obese comprises the underweight, normal, and overweight categories combined (Asia-Pacific classification for BMI [13] ).

BMI status*	n	%
Non-obese (underweight + normal + overweight)	648	91.9
Obese	57	8.1
Total	705	100.0

The association between socio-demographic characteristics and obesity status among adolescents is presented in Table 4. Obesity prevalence did not differ significantly across age groups (8.5% in 10-12 years, 7.8% in 13-15 years, and 8.1% in ≥16 years; Pearson's χ² = 0.076, p = 0.963). Similarly, no statistically significant association was observed between gender and obesity (9.0% in boys versus 7.1% in girls; Pearson's χ² = 0.806, p = 0.369; OR for boys versus girls 1.28, 95% CI 0.74-2.22). Although obesity prevalence was descriptively higher in Class I (10.7%) than in Classes II-V (8.0%, 7.7%, 5.7%, and 7.8%, respectively), the association between socioeconomic class and obesity was not statistically significant (Pearson's χ² = 2.192, p = 0.701).

**Table 4:** Association between socio-demographic factors and obesity status ^†^χ² (df) = Pearson chi-square statistic (degrees of freedom), testing the overall association between the variable and obesity status; one value applies to all categories of a variable. ^‡^OR = crude odds ratio for obesity (vs non-obesity), with 95% confidence interval (CI); the indicated reference (ref.) category was used as the comparator. ^§^Socio-economic class assigned using the Modified B.G. Prasad classification (2024 revision) [14]. ^‖^One or more cells had an expected count<5 in the overall contingency table; the chi-square result should be interpreted with caution, and Fisher’s exact test is recommended as a sensitivity check. Statistical significance was considered at p<0.05.

Variable	Category	Non-obese n(%)	Obese n(%)	χ² (df)^†^	p-value	OR (95% CI)‡
Age group (years)	10-12 (ref.)	161 (91.5)	15 (8.5)	0.076 (2)	0.963	1.00
	13-15	202 (92.2)	17 (7.8)			0.90 (0.44-1.86)
	≥16	285 (91.9)	25 (8.1)			0.94 (0.48-1.84)
Gender	Female (ref.)	313 (92.9)	24 (7.1)	0.806 (1)	0.369	1.00
	Male	335 (91.0)	33 (9.0)			1.28 (0.74-2.22)
Socio-economic class§	Class V (ref.)	47 (92.2)	4 (7.8)	2.192 (4)^‖^	0.701	1.00
	Class IV	100 (94.3)	6 (5.7)			0.70 (0.19-2.62)
	Class III	204 (92.3)	17 (7.7)			0.98 (0.31-3.04)
	Class II	172 (92.0)	15 (8.0)			1.02 (0.32-3.23)
	Class I	125 (89.3)	15 (10.7)			1.41 (0.45-4.47)

The associations between lifestyle factors and obesity status are presented in Table 5. Regular physical activity was significantly associated with obesity (χ² = 38.985, p<0.001; OR 5.14, 95% CI 2.94-8.97), with physically inactive adolescents showing a markedly higher prevalence of obesity (20.3%) than physically active adolescents (4.7%). Screen time was also significantly associated with obesity (χ² = 23.495, p<0.001), with obesity prevalence increasing progressively with longer daily screen time, from 0.0% among adolescents with no screen time to 18.2% among those reporting >3 hours/day. As no adolescent in the no-screen-time group was obese, a dose-response trend test was used instead of category-specific odds ratios, and this is confirmed that the odds of obesity rose steadily with each one-category increase in screen time (trend OR 1.87, 95% CI 1.43-2.43, p<0.001). Fruit consumption (χ² = 4.625, p = 0.032; OR 1.81, 95% CI 1.05-3.14) and vegetable consumption (χ² = 5.022, p = 0.025; OR 1.89, 95% CI 1.07-3.31) were both significantly associated with obesity, with obesity prevalence lower among consumers of fruit (6.6%) and vegetables (6.7%) than among non-consumers (11.4% and 12.0%, respectively). Trans-fat consumption was also significantly associated with obesity (χ² = 5.335, p =0.021; OR 1.98, 95% CI 1.10-3.56), with consumers showing a higher prevalence of obesity (10.2%) than non-consumers (5.4%). Effect sizes for all associations, expressed as odds ratios with 95% confidence intervals, are presented in Tables 4, 5.

**Table 5:** Association between lifestyle factors and obesity status ^†^χ² (df) = Pearson chi-square statistic (degrees of freedom), testing the overall association between the variable and obesity status; one value applies to all categories of a variable. ^‡^OR = crude odds ratio for obesity (vs non-obesity) with 95% confidence interval (CI); the indicated reference (ref.) category was used as the comparator. ^¶^No adolescent in the "None" screen-time category was obese (0/36); therefore, category-specific ORs were not estimated because of complete separation. Instead, a dose-response trend across ordered screen-time categories is reported. ^‖^One or more cells had an expected count <5 in the overall contingency table; the Chi-square result should be interpreted with caution. Statistical significance was considered at p<0.05.

Variable	Category	Non-obese n (%)	Obese n (%)	χ² (df)^†^	p-value	OR (95% CI)^‡^
Physical activity	Yes (ref.)	526 (95.3)	26 (4.7)	38.985 (1)	<0.001	1.00
	No	122 (79.7)	31 (20.3)			5.14 (2.94-8.97)
Screen time (hr/day)^¶^	None	36 (100.0)	0 (0.0)	23.495 (4)^‖^	<0.001	Trend OR (per 1-category increase) = 1.87 (1.43-2.43), p<0.001
	1	211 (95.9)	9 (4.1)			
	2	211 (93.4)	15 (6.6)			
	3	136 (86.6)	21 (13.4)			
	>3	54 (81.8)	12 (18.2)			
Fruit consumption	Yes (ref.)	453 (93.4)	32 (6.6)	4.625 (1)	0.032	1.00
	No	195 (88.6)	25 (11.4)			1.81 (1.05-3.14)
Vegetable consumption	Yes (ref.)	486 (93.3)	35 (6.7)	5.022 (1)	0.025	1.00
	No	162 (88.0)	22 (12.0)			1.89 (1.07-3.31)
Trans-fat consumption	No (ref.)	296 (94.6)	17 (5.4)	5.335 (1)	0.021	1.00
	Yes	352 (89.8)	40 (10.2)			1.98 (1.10-3.56)

## Discussion

This cross-sectional study, conducted among 705 school-going adolescents aged 10-19 years in Guwahati city, found that 57 (8.1%) of participants were obese; for descriptive context, 112 (15.9%) were overweight and 130 (18.4%) were underweight, and these two groups were classified as non-obese for the purpose of identifying factors associated with obesity. Bivariate analysis comparing obese with non-obese adolescents using the chi-square test showed that obesity was not significantly associated with age, gender, or socioeconomic class, but was significantly associated with physical activity, screen time, fruit consumption, vegetable consumption, and trans-fat consumption. The following sections interpret these findings in relation to the existing literature, consider possible explanations for the associations observed and not observed, and outline their implications for adolescent health policy and practice in urban India.

Prevalence of obesity and overweight

The prevalence of obesity (8.1%) and overweight (15.9%) observed in this study is broadly comparable to figures reported from other urban Indian settings, although direct comparison is complicated by the use of different BMI classification systems and age ranges across studies. Kaur et al. reported that obesity prevalence among school children in Delhi varied sharply with household income, ranging from 0.1% in low-income households to 6.8% in high-income households, with overweight prevalence rising from 2.7% to 15.3% over the same gradient [15]. Gupta et al. documented a secular rise in obesity prevalence among urban Indian adolescents, from 9.8% in 2006 to 11.7% in 2009, reflecting the rapid pace of the nutrition transition in Indian cities [16]. The obesity prevalence found in the present study (8.1%) sits within the range reported by these studies and reinforces the pattern of rising obesity among urban Indian adolescents over the past two decades.

At the same time, 18.4% of adolescents in this study were underweight, so that under-nutrition and over-nutrition coexisted within the same population - a pattern consistent with national data. The Comprehensive National Nutrition Survey (CNNS) reported a national thinness prevalence of 24.4% among Indian adolescents alongside a combined overweight/obesity burden of 5.9%, confirming that the two ends of the malnutrition spectrum commonly coexist within the same adolescent age group [17]. This dual burden reflects the uneven pace of nutritional transition in India, where pockets of continuing under-nutrition persist alongside a growing obesogenic environment, and underscores the need for school health screening protocols that identify both underweight and overweight adolescents rather than focusing on obesity alone.

Age and obesity

In contrast to several previous Indian studies, obesity prevalence in this study did not vary significantly across age groups (χ²=0.076, p=0.963); obesity was similarly distributed across the 10-12 year (8.5%), 13-15 year (7.8%), and ≥16 year (8.1%) groups. This finding differs from that of Rohilla et al., who reported a progressive rise in mean BMI from 19.23 kg/m² in the 10-13 year age group to 22.46 kg/m² in the 17-19 year age group among adolescents in an urban North Indian city [18], and from Ramachandran et al., who observed overweight prevalence increasing from 13.3% at 13 years to 18.9% at 18 years among urban Indian school children [19]. The absence of a significant age gradient in the present study may reflect the relatively narrow and overlapping age bands used for grouping (10-12, 13-15, and ≥16 years), the cross-sectional single-timepoint design, or cohort-level differences in growth patterns between school populations; it may also indicate that in this particular urban population, the accumulation of obesogenic exposures (dietary pattern, screen time, inactivity) occurs relatively early and remains fairly stable across the adolescent age range, rather than increasing progressively with age.

Gender and obesity

Obesity also did not differ significantly by gender in this study (χ²=0.806, p=0.369; OR for boys versus girls 1.28, 95% CI 0.74--2.22): 9.0% of boys and 7.1% of girls were obese. The absence of a significant gender difference in the present study is further supported by Table 2, which showed no statistically significant difference between boys and girls in mean height, weight, BMI, waist circumference, hip circumference, or waist-to-hip ratio (all p>0.05), suggesting that at the population level, boys and girls in this sample were exposed to broadly similar risk environments for obesity during this period of adolescence.

Socioeconomic status and obesity

Socioeconomic class, assigned using the Modified B.G. Prasad classification (2024 revision) [14], was not significantly associated with obesity in this study (χ²=2.192, p=0.701); as one or more cells in this five-category comparison had an expected count<5, this result should be interpreted with some caution, and a Fisher's exact test is recommended as a sensitivity check. Obesity prevalence was descriptively highest in Class I (10.7%) and lowest in Class IV (5.7%), with Classes II, III, and V showing broadly similar, intermediate prevalence (8.0%, 7.7%, and 7.8%, respectively); this pattern was not a clean monotonic gradient, in contrast to underweight prevalence, which rose consistently from 14.3% in Class I to 31.4% in Class V. The higher obesity prevalence observed in Class I relative to the lower classes is directionally consistent with a large body of Indian literature linking higher socioeconomic status with greater obesity risk. Bharati et al. reported 1.7 times higher odds of overweight/obesity among children from high socioeconomic status families compared with low socioeconomic status families in urban Wardha (95% CI: 1.3-2.3) [20], and Marwaha et al. found markedly higher obesity prevalence among children from upper socioeconomic strata compared with lower strata, both in boys (5.59% vs. 0.42%) and girls (5.73% vs. 0.28%) [21].

The failure of this pattern to reach statistical significance in the present study, together with the absence of a clean monotonic gradient, may reflect the relatively restricted socioeconomic range of a school-going sample compared with community-based samples that include out-of-school adolescents, the smaller cell sizes in the extreme classes (Class V, n=51, with only 4 obese) which reduce statistical power to detect an association across a five-category ordinal exposure, or a genuine narrowing of the socioeconomic gradient in obesity risk as smartphone-based sedentary behaviour and processed food access become more evenly distributed across socioeconomic strata in urban India. Given the higher descriptive prevalence observed in Class I, socioeconomic class should not be dismissed as a variable associated with adolescent obesity in this setting; a larger, adequately powered study may be needed to confirm whether this association is genuinely absent or whether the present study was underpowered to detect it.

Physical activity and obesity

Physical activity showed a statistically significant association with obesity (χ²=38.985, p<0.001). Adolescents who did not engage in regular physical activity had a markedly higher prevalence of obesity (20.3%) compared with those who were physically active (4.7%) - more than four times the burden of obesity among the physically inactive, corresponding to just over five times the crude odds of obesity among the physically inactive (OR 5.14, 95% CI 2.94--8.97). This represents the strongest bivariate association observed in this study and is consistent with the well-established protective role of physical activity against adolescent obesity.

This finding aligns with Bharati et al., who reported that adolescents who engaged in regular physical activity had approximately 40% lower odds of obesity than inactive adolescents (95% CI: 0.41-0.87, p=0.008) in a study conducted in Central India [20], and with Moitra et al., who found that Indian adolescent boys with low physical activity (<60 minutes/day of moderate-to-vigorous activity) had 2.10 times higher odds of overweight/obesity, with the odds rising further when combined with excess screen time [22]. Given the consistency of this association across settings, structured physical activity remains one of the most actionable targets for school-based obesity prevention in this population.

Screen time and obesity

Screen time showed a strong association with obesity (χ²=23.495, p<0.001), with a clear dose-response gradient: obesity prevalence rose from 0.0% among adolescents reporting no daily screen time, to 4.1% among those with 1 hour/day, 6.6% with 2 hours/day, 13.4% with 3 hours/day, and 18.2% among those reporting more than 3 hours/day. Notably, no adolescent in the no-screen-time group was classified as obese, whereas nearly one in five adolescents (18.2%) in the highest screen-time category was obese; given the zero observed obese cases in the no-screen-time group (expected count<5), category-specific odds ratios were not computed for this variable; instead, a dose--response trend odds ratio estimated by grouped logistic regression across ordered screen-time categories was 1.87 (95% CI 1.43--2.43) per one-category increase in exposure (p<0.001), quantifying the graded gradient evident in the prevalence figures, and this association should be interpreted with some caution at the lowest exposure level despite the clear overall gradient. In simple terms, this trend test showed that each one-step increase in daily screen-time category (for example, moving from 1 hour/day to 2 hours/day) was linked to roughly 87% higher odds of obesity, giving statistical confirmation of the step-by-step rise in obesity seen in the raw percentages above.

This dose-response pattern is consistent with the international literature. Katzmarzyk et al., in a 12-country study, found that each additional hour of daily screen time was associated with a 15% increase in the odds of obesity (95% CI: 1.08-1.23) [23]. In a nationally representative United States sample, Bakour et al. reported that adolescents with ≥4 hours/day of screen time had more than double the odds of overweight/obesity compared with those with<1 hour/day (adjusted OR: 2.19, 95% CI: 1.73-2.77) [24]. The magnitude and consistency of the dose-response relationship observed here suggest that screen time is one of the most important modifiable behavioural targets for adolescent obesity prevention in this population, and that interventions may need to specifically address graded reductions in screen exposure rather than a single threshold.

Dietary habits and obesity

Both fruit and vegetable consumption were significantly associated with obesity in this study. Fruit consumption showed a significant association (χ²=4.625, p=0.032; OR 1.81, 95% CI 1.05--3.14), with non-consumers of fruit having close to twice the crude odds of obesity of consumers, and obesity prevalence lower among fruit consumers (6.6%) than non-consumers (11.4%). Vegetable consumption was also significantly associated with obesity (χ²=5.022, p=0.025; OR 1.89, 95% CI 1.07--3.31), with vegetable consumers showing a lower obesity prevalence (6.7%) than non-consumers (12.0%). These findings are consistent with Ledoux et al.'s systematic review, which found that higher fruit and vegetable intake was generally, though not uniformly, associated with lower adiposity in children and adolescents [25], and with Faizi et al., who reported markedly higher odds of overweight/obesity among adolescents who did not consume vegetables in a nonmetropolitan Indian city (OR=5.13, 95% CI: 3.34-7.88) [26]. Restricting the outcome to obesity alone, rather than the combined overweight/obese category, revealed a significant fruit-consumption association that was not apparent when overweight and obesity were analysed together, suggesting that fruit intake may be more closely linked to the more extreme end of the BMI distribution than to milder excess weight; a more granular dietary assessment tool capturing frequency, quantity, and variety of intake may help clarify the strength and consistency of both associations in future studies in this population.

Trans-fat consumption was also significantly associated with obesity (χ²=5.335, p=0.021; OR 1.98, 95% CI 1.10--3.56), with consumers having close to twice the crude odds of obesity of non-consumers, and showing a higher prevalence of obesity (10.2%) than non-consumers (5.4%). This finding is consistent with the broader literature on trans fatty acids and adiposity; Thompson et al., in a systematic review, concluded that there is limited but consistent evidence from epidemiological studies that higher intake of industrial trans fatty acids contributes to greater weight gain [27]. In this study, more than half of adolescents (392/705, 55.6%) reported consuming trans-fat-containing foods, reflecting the widespread availability of processed snacks, fried foods, and bakery products in the urban Indian food environment, and pointing to a specific, modifiable dietary target for obesity prevention efforts in this setting.

Strengths and limitations

This study has several strengths, including a reasonably large sample size (n=705) drawn through stratified random sampling from both government and private schools, which enhances the representativeness of the findings for the urban school-going adolescent population of Guwahati; standardized anthropometric measurement procedures; the assessment of multiple potentially modifiable lifestyle exposures alongside sociodemographic characteristics; and the quantification of each bivariate association using odds ratios with 95% confidence intervals in addition to chi-square testing, which conveys the magnitude of association rather than statistical significance alone.

Several limitations should also be acknowledged. First, the study classified obesity using fixed Asia-Pacific BMI cut-offs rather than age- and sex-specific WHO BMI-for-age reference standards recommended for adolescents. This represents a considered trade-off rather than an oversight: comparisons of growth references among Indian school-going populations indicate that the WHO 2007 standard does not systematically underestimate overweight/obesity relative to other commonly used references, whereas some national or IOTF-based criteria can. For instance, a study of affluent school students aged 8-18 years in Rajkot, Gujarat, found obesity prevalence of 14.0% by IAP 2015 criteria and 11.1% by WHO 2007 criteria, compared with only 5.1% by IOTF criteria, illustrating that the choice of reference can shift point prevalence several-fold within the same population [28]. Similarly, a comparison of growth references among school-going youth in Delhi concluded that the WHO reference did not underestimate obesity relative to IOTF criteria and remained suitable for tracking national and international trends [29]. Because the individual-level age, sex, height, and weight data required to compute WHO BMI-for-age z-scores were not available in a form permitting re-analysis for this manuscript, a direct reclassification of the present cohort using WHO criteria was not possible. However, the direction of the discrepancy reported in comparable Indian adolescent populations suggests that adopting WHO BMI-for-age criteria would be unlikely to substantially lower the obesity prevalence reported here, and could in some age-sex strata modestly raise it. The Asia-Pacific criteria were nonetheless retained because of their established use in the Indian literature and their greater sensitivity for identifying obesity-related cardiometabolic risk in Asian populations at lower absolute BMI values; their use may still have resulted in some misclassification of nutritional status among individual adolescents and limits direct numerical comparability with studies using WHO BMI-for-age criteria. Notably, fixed Asia-Pacific or adult-equivalent BMI cut-offs applied across the same broad 10-19 year adolescent age range, rather than age- and sex-specific WHO BMI-for-age z-scores, are not unique to the present study but are precedented within several of the very studies against which the present prevalence estimates are compared, including Gupta et al. [16] (adolescents 14-17 years, Delhi) and Rohilla et al. [18] and Ramachandran et al. [19] (adolescents 10-19 years and urban Indian school children, respectively); this shared methodological approach across studies somewhat mitigates, though does not eliminate, the comparability concern raised above.

Second, the clustered school-based sampling design was not accounted for in the statistical analysis. Because students within the same school may share environmental and socioeconomic characteristics, conventional chi-square tests, which assume independent observations, may yield overly precise (narrow) significance estimates. Future analyses should consider design effects or clustering-adjusted methods (e.g., multilevel models or robust/clustered standard errors) to obtain valid estimates of statistical significance.

Third, no multivariable analysis was performed in this study. Physical activity, screen time, dietary behaviours, socioeconomic status, and age may confound one another, and consequently, none of the variables examined can be described as an independent risk factor for adolescent obesity based on the present analysis. Multivariable modelling would be required to disentangle these interrelationships and to identify independent predictors.

Fourth, several bivariate tests were performed without adjustment for multiple comparisons. Therefore, some statistically significant findings may represent chance associations and should be interpreted cautiously, particularly because no adjusted effect estimates or prespecified multiplicity correction was applied. These findings should be considered exploratory and require confirmation in future studies.

Fifth, although schools were selected using stratified random sampling, only seven schools were included, and fixed numbers of students were recruited from each selected school rather than allocating participants in proportion to the total enrolment of each school. Consequently, the study sample may not fully represent the distribution of adolescents across all schools in Guwahati, and the generalizability of the findings should therefore be interpreted with caution.

Sixth, students who were absent on the day of data collection were replaced by the next eligible student on the attendance register in order to achieve the predetermined sample size. This approach may have introduced selection bias, as students who were absent from school may have differed systematically from those who were present with respect to health status, lifestyle behaviours, or other characteristics.

Seventh, sampling weights were not applied during the analysis. Because the number of participants selected from the different school strata was not based on formal weighting procedures, the prevalence estimates reported in this study may not fully represent the true distribution of the entire school-going adolescent population of Guwahati.

Finally, although the data collection instrument was pretested for clarity and comprehensibility, it was not subjected to formal validity or reliability testing. This may have affected the precision of some self-reported variables, particularly screen time and dietary intake, which are also inherently susceptible to recall bias and social desirability bias.

## Conclusions

The present study demonstrated that 8.1% of school-going adolescents in Guwahati were obese; for context, 15.9% were overweight, and 18.4% were underweight, indicating the coexistence of over-nutrition and under-nutrition in this population. On bivariate analysis comparing obese with non-obese adolescents, obesity was not significantly associated with age, gender, or socioeconomic class, but was significantly associated with physical activity, screen time, fruit consumption, vegetable consumption, and trans-fat consumption, with physical inactivity carrying the largest crude odds of obesity (OR 5.14, 95% CI 2.94-8.97) among the factors examined.

The observed associations suggest that promoting physical activity, reducing recreational screen time, and encouraging healthy dietary practices may be beneficial for improving adolescent health. However, as this was a cross-sectional study, causal relationships cannot be established. Longitudinal studies with multivariable analyses and appropriate adjustment for school-level clustering are required to confirm these associations.

## Appendices

**Table 6:** Operational definitions of key lifestyle and behavioural variables

Variable	Operational Definition
Regular physical activity	Assessed using the item “Are you involved in any kind of physical activity?” (Yes/No), consistent with the World Health Organization’s definition of physical activity as any bodily movement produced by skeletal muscles requiring energy expenditure, including activity during leisure time, transport, or occupational/academic routine. Participants reporting any such activity were classified as “Yes” (physically active/regular physical activity); those reporting none were classified as “No.”
Screen time	Screen time referred to recreational use of television, smartphones, tablets or computers during a typical weekday and excluded school-related activities. Total self-reported average daily time spent on screen-based devices (television, mobile phone, computer/tablet), captured via the item “How much time do you usually spend on screen time?” with response options of None, 1, 2, 3, or more than 3 hours/day. Analysed as a 5-level ordinal variable.
Fruit intake	Assessed using a food-frequency questionnaire, with response options of “never,” “<1 time per month,” “1–3 times per month,” “1–2 times per week,” “3–6 times per week,” or “daily” (estimated serving size of ½ cup). “Never” was classified as “No (non-consumer)”; all other response options, including “<1 time per month,” were classified as “Yes (consumer),” consistent with standard practice of treating any reported intake as consumption.
Vegetable intake	Assessed using the same food-frequency response options as fruit intake. “Never” was classified as “No (non-consumer)”; all other response options were classified as “Yes (consumer).”
Trans-fat intake	Assessed using a food-frequency item covering fried foods, processed/packaged foods, and bakery products, with the same response options as above. “Never” was classified as “No (non-consumer)”; all other response options were classified as “Yes (consumer).”
Substance use	Composite variable comprising four individually assessed items: tobacco chewing, cigarette/bidi smoking, smokeless tobacco use, and alcohol consumption. Current tobacco use (smoked or smokeless) was defined as use above a minimum lifetime threshold (≥100 cigarettes for smoked tobacco; ≥10 instances for smokeless tobacco) with continuing use within the preceding 6 months; current alcohol use was defined as consumption of at least one alcoholic drink within the preceding year. Participants reporting current use of any one or more of these four substances were classified as “Yes”; those reporting none were classified as “No.”
